# Developing a Culturally Sensitive ICF-Based Tool to Describe Functioning of Children with Autism Spectrum Disorder: TEA-CIFunciona Version 1.0 Pilot Study

**DOI:** 10.3390/ijerph18073720

**Published:** 2021-04-02

**Authors:** Silvana B. Napoli, María Paula Vitale, Pablo J. Cafiero, María Belén Micheletti, Paula Pedernera Bradichansky, Celina Lejarraga, Maria Gabriela Urinovsky, Anabella Escalante, Estela Rodriguez, Verónica Schiariti

**Affiliations:** 1 Division of Interdisciplinary Clinics, Child Developmental Pediatric Unit, Children Hospital JP Garrahan, Buenos Aires C 1245 AAM C.A.B.A, Argentina; mpauvitale@gmail.com (M.P.V.); pcafi67@gmail.com (P.J.C.); belenmicheletti@gmail.com (M.B.M.); paupeder1@gmail.com (P.P.B.); celileja@gmail.com (C.L.); gabrielaurinovsky@gmail.com (M.G.U.); ase.escalante@gmail.com (A.E.); rodriguez.estela15@gmail.com (E.R.); 2 Division of Medical Sciences, University of Victoria, Victoria, BC V8W 2Y2, Canada; vschiariti@uvic.ca

**Keywords:** ICF, autism spectrum disorder, functioning, child, measure, assessment

## Abstract

Background: Autism spectrum disorder (ASD) affects the daily functioning of children and their families; however, in Argentina, there are no standardized tools to guide the description, evaluation, and follow-up of functioning and disability of children with ASD. To fill this gap, the overarching purpose of this study was to create a novel tool guided by the International Classification of Functioning, Disability, and Health (ICF) Core Sets for ASD for clinical practice. Methods: A multistep methodology was used to identify the most relevant ICF categories for an Argentinian clinical setting. The content of this ICF-based shortlist was piloted and revised according to the results. Subsequently, a toolbox of measures was proposed to operationalize each ICF category. Finally, profiles of the functioning of 100 children with ASD were created. Results: An ICF-based tool called TEA-CIFunciona was created, consisting of 32 ICF categories (10 body functions, 15 activities and participation, 7 environmental factors categories). The application of TEA-CIFunciona incorporated a family-centered approach in ASD evaluations and helped identify functional needs. Conclusions: TEA-CIFunciona is the first ICF-based instrument that guides the description of functioning of children with ASD in Argentina. TEA-CIFunciona standardizes collaborative assessments in pediatric ASD populations in Latin American contexts.

## 1. Introduction

Autism spectrum disorder (ASD) is a developmental disorder with a variable phenotypic expression characterized by communication and socialization difficulties and repetitive and stereotyped patterns of behavior [1,2]. ASD is generally diagnosed in early childhood and is considered to be secondary to an alteration in early brain development and neural reorganization [3]. The core symptoms of ASD have an early onset in childhood and tend to persist throughout the lifespan.

The global estimated prevalence of ASD is 1–2% [4]. In Argentina there is a lack of data on the overall prevalence of ASD; however, isolated studies have found similar figures to the prevalence estimates for the Americas by the World Health Organization (WHO) 0.7% [5,6].

Early diagnosis of ASD guides individualized early intervention. ASD diagnostic assessment warrants a detailed evaluation of the behavioral features described in manuals of diagnosis and classification, including the Diagnostic and Statistical Manual of Mental Disorders (DSM-5) [7] and the International Statistical Classification of Diseases and Related Health Problems 10th revision [8]. However, a timely diagnosis of ASD should be complemented with a comprehensive assessment of functional needs performing everyday life activities, to ensure meaningful and adequate interventions.

In 2001 the WHO proposed the use of the International Classification of Functioning, Disability, and Health (ICF) [9] to describe functioning and disability from a biopsychosocial perspective. *Functioning* is an umbrella term to describe what a person with a health condition does or is able to do in everyday life at home, school, and in the community [9,10]. In 2007 a Child and Youth version of the ICF (ICF-CY) [11] was published specifically to capture functioning in developing individuals by adding and expanding on the descriptions of categories provided in the ICF. The ICF-CY facilitates the description of functional *abilities* and limitations in each area of development, promoting a family/child-centered approach [11]. This is important because children with neurodevelopmental disorders and their families cherish the child’s functional abilities rather than the physical challenges, limitations, and participation restrictions associated with a specific diagnosis [12].

Moreover, the ICF systematically incorporates the role of environmental factors, including family, friends, therapists, societal attitudes, services, health systems and policies, and products and technology, as essential elements that facilitate or hinder participation and social inclusion [9,11]. Additionally, all content in the ICF is in conformity with international conventions and declarations on the rights of children and persons with disabilities, encouraging a human rights-based approach [9,11].

The ICF structures health and health-related domains into a hierarchy starting with components, then chapters, followed by categories. An ICF category is represented by an alphanumeric code. This code contains a letter that denotes one of the components of the ICF: body functions (b), body structures (s), activities and participation (d), and environmental factors (e) [9,11]. The component index letters are followed by a numeric code starting with the chapter number adding one digit (e.g., b**1** mental functions), followed by a second-level category code adding two digits (e.g., b1**67** mental functions of language), and third and fourth level code by adding one digit respectively (e.g., b167**0** reception of language and b1670**0** reception of spoken language). The categories with their corresponding codes must be completed with a qualifier: one, or more numbers after a point which denotes the severity of the problem or the extent to which a factor is a facilitator or barrier [9].

A key contribution of the ICF is to provide a framework and a structure for collecting and organizing clinical information evaluated by professionals worldwide, providing a universal language [9,11]. As such, the ICF is an important contribution to the growing interest in identifying children’s needs based on their profiles of functioning rather than using only diagnostic labels, including ASD [13,14,15]. 

The practical application of the ICF-CY (from here we use ‘ICF’ to refer to both classifications) has been a challenge in clinical practice, including in clinical assessments of children with ASD, as the entire classification is comprehensive, consisting of 1685 ICF categories, In 2018, Bölte et al. following a rigorous step-wise multiple study methodology specified by the ICF Research Branch of the WHO Collaborating Centre for the Family of International Classifications in Switzerland, developed ICF Core Sets for ASD to facilitate its use [16]. ICF Core Sets represent shortlists of ICF categories that cover the most relevant areas of functioning and disability in a specific condition, which facilitate the application of the ICF in day-to-day practice. The Comprehensive Core Set for ASD consists of 111 categories and the common abbreviated set has 60 categories; the version for children 0–5 years of age has 73 categories, the version for children 6–16 years of age 81 categories, and the version for adults 79 categories [16,17].

Even though the ICF Core Sets for ASD, developed for the international context, highlight the most relevant categories for ASD out of the entire ICF classification, the length and complexity of these ICF Core Sets make them still difficult to use in everyday clinical encounters in Argentina.

There are multiple benefits of adopting ICF-based tools in clinical practice [15,18]. The systematic use of ICF-based tools defining the minimal key areas of functioning to be measured and reported for a given condition may be helpful to guide treatment planning, to identify facilitators and environmental barriers, and to reduce disparities in services. Moreover, ICF-based tools facilitate involvement of parents as active participants in the decision-making process on treatment goals and evaluation of intervention outcomes.

In Argentina, there is consensus on the diagnosis and treatment of people with ASD [19], but there are no guidelines that standardize the assessment of daily functioning in children with ASD. This highlights the need of a tool to systematically describe functioning of children with ASD in a comprehensive way. To fill this gap, the overall purpose of this study was to create a brief ICF-based tool to standardize assessments of ASD, with the following specific aims: (1) to identify the most relevant categories from the ICF Core Sets for ASD in order to describe the daily functioning of children with ASD in our country; (2) to propose measurement scales to evaluate each ICF category identified in aim 1; (3) to assess the feasibility of using a self-developed shortlist of ICF categories in clinical encounters; and finally (4) to describe the profile of the functioning of children with ASD at our national referral center.

## 2. Materials and Methods

The study is a descriptive cross-sectional design with prospective data analysis conducted between January and December 2019. Study data were collected and managed using REDCap electronic data capture tools hosted at Prof. Dr. Juan P. Garrahan” Hospital (JPGH). For statistical analysis, RStudio Software was used. The study was approved by the Ethical Review Board of JPGH and a written informed consent form was signed by parents.

### 2.1. Setting

The Argentinian health care system is characterized by considerable fragmentation, including a public system, work-related social insurances, and private health insurances, which causes great heterogeneity in assessments and interventions of ASD. According to the latest national census data, 36% of the population depends on publicly-funded medical insurance [20], showing the essential role of public hospitals in service provision in the country. Hence, in Argentina, there is increasing demand for providers of health services for persons with ASD in the public system, but only a few exist. One of these service providers is “Prof. Dr. Juan P. Garrahan” Hospital, the largest national public pediatric referral center in Buenos Aires, Argentina. The Division of Interdisciplinary Clinics provides consultations for the evaluation, diagnosis, and management of children with neurodevelopmental disabilities. Our division receives approximately 5000 visits per year. Out of those visits, approximately 120 visits per year are related to ASD diagnosis and a similar number to ASD follow-up visits, this reflects the average over the last three years.

### 2.2. Study Team—Professionals and Caregivers

Our interdisciplinary team consists of developmental pediatricians (*n* = 6), speech and language therapists (*n* = 4), and special educators (*n* = 6), with more than 15 years of experience in the diagnosis and follow-up of children with ASD. In addition, the study was conducted with active collaboration from the parents and caregivers of children with ASD attending follow-up visits at our clinic.

### 2.3. Study Participants 

Children and their caregivers who attended regular ASD follow-up visits at the Child Developmental Pediatric Unit of the Division of Interdisciplinary Clinics at JPGH, were invited to participate, those who provided consent were included in a consecutive sampling. The inclusion criteria were a child with a diagnosis of ASD made at the Division of Interdisciplinary Clinics and age of the child under 16 years old. There were no exclusion criteria.

Children’s information including gender, age, comorbidities, genetic condition, or another neurodevelopmental disorder were collected from health records. Severity of ASD was categorized following the DSM-5 levels of severity [7]. An unsatisfied basics needs (UBN) [20] score, measuring structural poverty was assigned to describe sociodemographic characteristics.

### 2.4. Procedure

#### 2.4.1. Interprofessional and Family-Centered Approach

The overall purpose of this study was to create a brief culturally sensitive ICF-based tool to standardize assessments of functioning of children with ASD, especially to describe functioning in terms of performance at home, at school, and in the community. In every step of the study, the interprofessional study team, along with parents and caregivers, maintained active communication and collaborated sharing their different perspectives on day-to-day functioning. Thus, a multistep methodology was used to achieve the overall and specific aims, as follows:

- Aim (1) Identify the most relevant categories from the international ICF Core Sets for ASD to describe the daily functioning of children with ASD.

During two months, we conducted weekly sessions to familiarize ourselves with the content of the ICF classification and structure. We reviewed the literature related to the development and content of the comprehensive ASD Core Set [16,17]. In addition, electronic health records were reviewed to capture the main concerns described by families of children with ASD in previous years. Using the main concerns, each team member completed a checklist linking the meaningful concepts to the ICF categories included in the comprehensive ASD Core Set. We followed the ICF linking guidelines proposed by Cieza et al. [21]. ICF categories with an agreement of 75% or greater were included in our shortlist. This ICF-based shortlist was called TEA-CIFunciona.

- Aim (2) Propose measurement scales to evaluate each ICF category included in TEA-CIFunciona.

During the following month, we conducted weekly sessions where measurement tools were sought for each category included in our shortlist. We created a toolbox of measures to assess the categories in TEA-CIFunciona. Specifically, we tried to identify a one-to-one correspondence between an item of a measure and single ICF categories, at times this was not possible. Therefore, we proposed a set of items. When we could not identify a scale or measure to cover the content of the ICF category, we developed questions to assess that category. We ensured that the content of the questions represented the ICF category, using the ICF linking guidelines proposed by Cieza et al. [21].

- Aim (3) Feasibility and psychometric properties of TEA-CIFunciona version 0.0.

TEA-CIFunciona version 0.0 was piloted to check face validity, reliability, and feasibility in 20 patients. Interviews were conducted by two members of the study team who independently applied TEA-CIFunciona. Face validity: A questionnaire was answered by both evaluators to determine if all aspects of the consultations were included in TEA-CIFunciona. In addition, a questionnaire was answered by the parents to ensure that all their concerns were addressed; each family was additionally asked to give their opinion about the interview.

Inter-rater reliability: Two evaluators participated in each interview and independently scored all ICF categories included in TEA-CIFunciona. Cohen’s Kappa statistics were used to evaluate inter-rater reliability. *k* of 0.61–0.80 are considered as substantial agreement and *k* > 0.80 as excellent agreement. Categories with a Kappa > 0.60 were maintained on our shortlist. Feasibility: raters were asked to provide feedback on usability, clarity, and objectivity of the TEA-CIFunciona using a brief questionnaire.

- Aim (4) Describe the profile of functioning of children with ASD using TEA-CIFunciona.

Finally, using the final version of TEA-CIFunciona (version 1.0), we described the profile of functioning of a larger group of children with ASD (*n* = 100). We translated clinical information into qualifiers following a self-developed guide, see below.

#### 2.4.2. Translating Clinical Information and Standardized Assessment into ICF Qualifiers

As there is no gold standard to guide the translation of clinical information into ICF qualifiers, we created a guide for our pediatric clinical setting. First, we identified the best content correspondence [21] between item/s from standardized measures used in our ASD clinic and the content of the ICF categories included in TEA-CIFunciona. Then, we followed the ICF generic scale for problem severity (0–4% no problem/5–24% mild problem/25–49% moderate problem/50–95% severe problem/96–100% complete problem) [9,11] and compared it to the grading system of the assessment tools included in the toolbox. Additionally, we coded clinical information directly into ICF categories and qualifiers using our clinical expertise. Some categories were evaluated directly by parents, using the visual analog scale (VAS) as reported in other studies [22], including a visual response card created by the researchers to facilitate parents’ understanding when identifying the degree of difficulty experienced by the children in a category. Parents’ answers were directly translated into ICF qualifiers. Supplementary Material S1 shows examples of how clinical information and scores from standardized measures were translated into the ICF qualifiers.

## 3. Results

**Figure 1:**
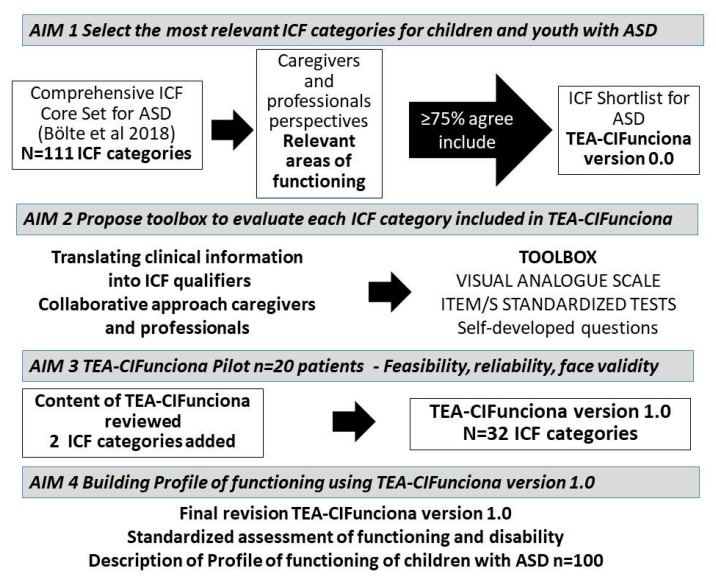
Multistep process for the development of TEA-CIFunciona and its clinical application.

The multistep process led to the creation of TEA-CIFunciona version 1.0 and subsequently its application in clinical practice (Figure 1). TEA-CIFunciona version 1.0 consists of 32 ICF categories including, 10 body functions, 15 activities and participation, and 7 environmental factors.

### 3.1. Aim (1) Identify the Most Relevant Categories from the ICF Core Sets for ASD in Order to Describe the Daily Functioning of Children with ASD

Table 1 shows the categories included in TEA-CIFunciona version 1.0 as well as the toolbox to operationalize each category. All ICF categories were obtained from the Comprehensive Core Set for ASD, except d815 preschool education. This category was included to cover a common concern expressed by parents during clinical consultations.

### 3.2. Aim (2) Propose Measurement Scales to Assess Each ICF Category. Development of a Toolbox

All selected scales are used regularly in assessments of ASD, and the study team is trained in their use. The selection of the tools was based on consensus. From each tool we selected the item/s that best covered the content of the ICF category (Table 1). Experts from other national and international institutions were consulted to provide feedback on the proposed toolbox.

### 3.3. Aim (3) Feasibility and Psychometric Properties of TEA-CIFunciona

We showed that TEA-CIFunciona was reliable, relevant, and feasible for application in our busy clinical consultations. After we piloted the first version of TEA-CIFunciona, we revised the tool based on parents’ and professionals’ feedback. Hence, two categories were added, as follows: Individual attitudes of people in positions of authority (e430) and Associations and organizational services, systems, and policies (e555). Two categories (b125 and b140) showed a lower level of agreement than expected; therefore, it was agreed to prioritize parents’ opinion and they scored the categories using the VAS.

### 3.4. Aim (4) Describe the Profile of Functioning of Children with ASD Using TEA-CIFunciona; Clinical and Demographic Variables of the Sample

Table 2 shows the general characteristics of the sample using the TEA-CIFunciona version 1.0. Overall, 100 children were assessed, 81% were boys, with a mean age of 7 years and 4 months (range, 3 to 16 years), 10% had structural poverty (positive UBN score), and 20% lacked health insurance coverage. Almost 69% presented with an associated medical condition (e.g., sleep disorder, obesity, epilepsy) and 70% with associated developmental disorder (e.g., intellectual disability, developmental coordination disorder). Importantly, we included a representative sample of all severity levels for ASD proposed by the DSM-5 [7].

### 3.5. Profile of Functioning Using TEA-CIFunciona Version 1.0 (n = 100 Children with ASD)

Table 3 shows the frequency of the impairments, limitations, and restrictions in daily functioning in the corresponding components of body functions, activities and participation, and environmental factors. In the component body functions, intrapersonal functioning (b125) was considered to be a moderate or severe problem in 61% of the children. A moderate-to-complete problem was observed in reception of language (b1670) in 45% and in expression of language (b1671) in 73%.

In activities and participation, almost 20% of caregivers reported that their children had severe problems in relation to toileting (d530) and eating (d550). Education, considering school and preschool education (d815 and 820), appeared to be a problem in 40% of the sample, with different levels of severity. Speaking (d330) and conversation (d350) were considered moderate to complete problems in 33% and 79% of the children, respectively. Maintaining a relationship (d720 and d7500) and the use of leisure time (d920) were common functional challenges.

Regarding environmental factors, although the majority of parents considered attitudes of people in positions of authority (e430) a facilitator, many (24%) found it to be a barrier. Immediate family (e310) was a facilitator in 97% and health professionals (e355) in 61% of the sample.

Parents most often described problems in dispositions and intra-personal functions (b125), attention functions (b140), perceptual functions (b156), and eating (d550). Overall, support from parents’ associations (for example NGOs) was seen as facilitators but appear to be an underused resource for families. 

**Figure 2:**
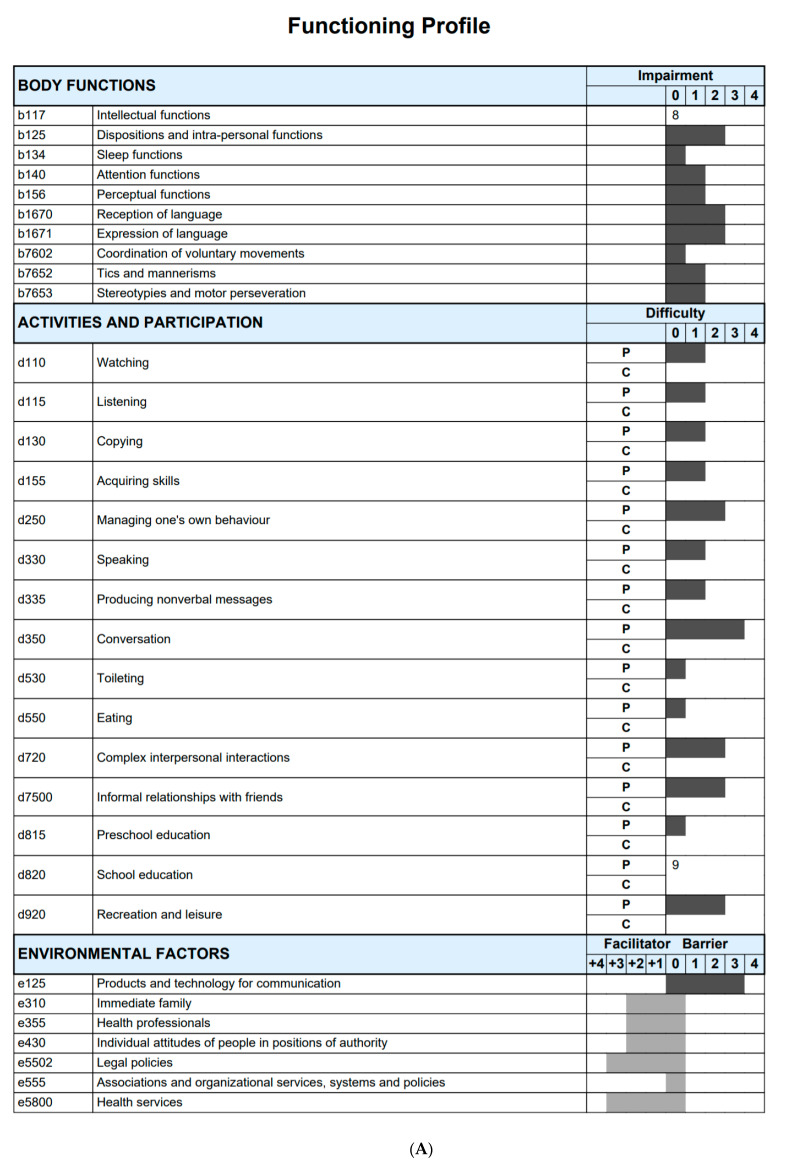

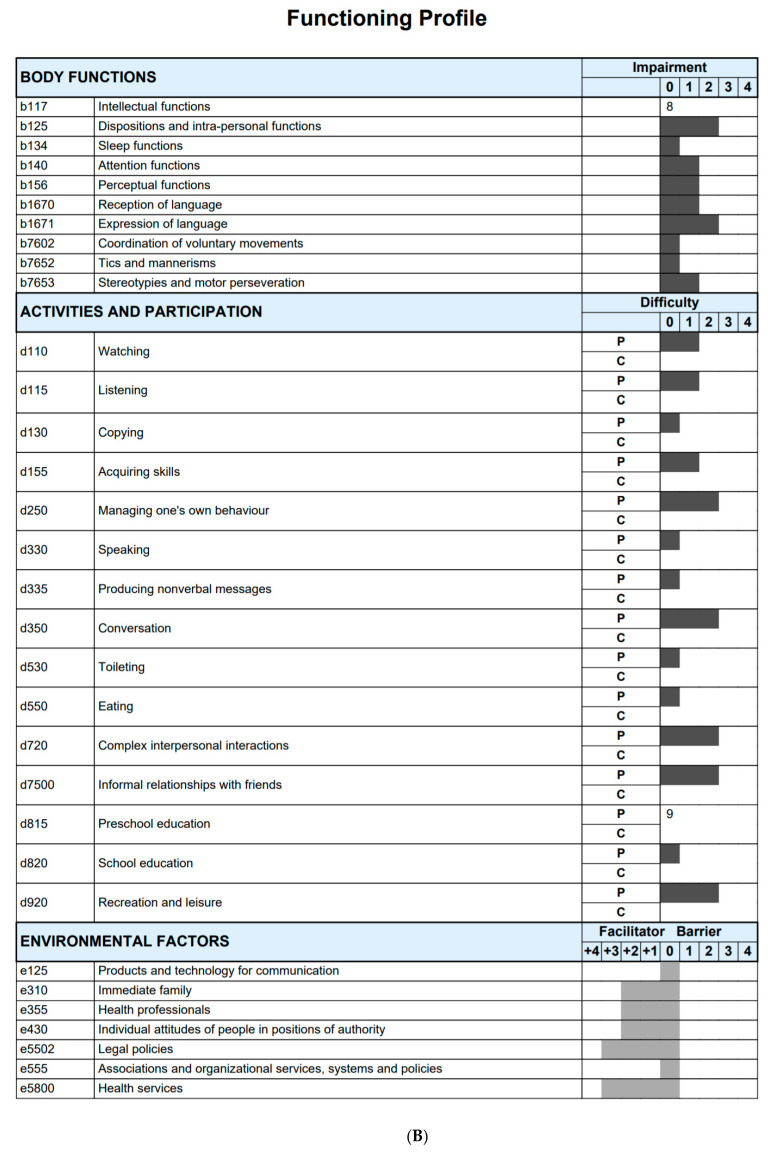
(**A**) Profile of functioning of children with ASD (autism spectrum disorder) < 6 years of age using TEA-CIFunciona version 1.0. (**B**) Profile of functioning of children with ASD 6 to 16 years of age using TEA-CIFunciona version 1.0.

Figure 2A,B summarize the profiles of functioning of children with ASD under 6 years and ≥6 to 16 years, using TEA-CIFunciona version 1.0. As shown, there are many commonalities among the groups; however, each age-group shows unique functional characteristics and environmental factors, for example, in areas of receptive language, coordination of movements, copying skills, speaking, producing non-verbal messages, conversation, and using or accessing products and technologies for communication. Using TEA-CIFunciona, we were able to show that younger children with ASD have greater impact on functioning and less access to products and technology for communication. This information can guide interventions as well as modifications of the environment.

ICF Qualifiers in body functions, body structures and activities and participation: 0 = no problem; 1 = mild problem; 2 = moderate problem; 3 = severe problem; and, 4 = complete problem. ICF Qualifiers in environmental factors: 0 = no barrier/facilitator; +1 = mild facilitator; +2 = moderate facilitator; +3 = substantial facilitator; +4 = complete facilitator; 1 = mild barrier; 2 = moderate barrier; 3 = substantial barrier; and 4 = complete barrier. The component personal factors (pf) does not have ICF categories assigned, therefore it is recommended to add themes representing personal factors to complement the profile of functioning.

The ICF qualifiers used to create the profile of functioning of this study sample represent the qualifiers that have the highest percentage within each ICF category. This profile of functioning was built using the ICF-based documentation form on this web page https://icf-core-sets.org/es/page0.php (accessed on 1 April 2019), courtesy ICF Research Branch. P (performance): describes what an individual does in his or her current environment. C (capacity): describes an individual’s ability to execute a task or an action, meaning the highest probable level of functioning that a person may reach in a “standardized” environment.

## 4. Discussion

This study describes the creation of a novel ICF-based instrument called TEA-CIFunciona version 1.0. This is the first study conducted in Latin America that describes functioning in children with ASD. The contributions of this study are multiple, as follows: (1) identification of the most relevant areas of functioning and disability to standardize assessments of children with ASD in Argentina; (2) proposing a toolbox with standardized scales and questionnaires to operationalize the categories selected; (3) description of the profile of functioning of a large sample of children with ASD (*n* = 100) in the region using the ICF universal language.

### 4.1. Benefits of Using TEA-CIFunciona Version 1.0

TEA-CIFunciona incorporates the most relevant ICF categories for our cultural and pediatric setting. The application of TEA-CIFunciona helped us to incorporate a family-centered approach in the clinic. Consideration of parental views shifted the focus of follow-up visits towards the aspects of daily living significant to them. This modified the team’s perspective; thus, parents’ point of view and concerns were prioritized while taking into account the available resources.

In addition, the recognition of children’s strengths and abilities was highly appreciated by the parents. For example, use of eating utensils may not be important to the practitioner, but may be essential to them. Generally, these skills can only be appreciated by the professional after a thorough investigation of everyday activities [23]. 

The systematic consideration of environmental characteristics allows us to draw some general conclusions regarding the difficulties these children and their families must overcome, and which support they considered most important. In most cases, the immediate family was identified as a substantial or complete facilitator, and its absence as an important barrier. This confirms once again not only the importance of a strong family structure, but also the need for support networks in the community and the notion that clinical interventions are successful when the uniqueness and diversity of families are recognized [24].

Another finding was that the pediatrician was identified as a facilitator in only 61% of the cases. The reasons given for this rating were lack of knowledge on ASD, or negative attitude. Often children with ASD do not have a primary care pediatrician and parents navigate a fragmented health care system without guidance. Sometimes the pediatrician is unaware of the family’s priorities, and whether treatments or supports are working. Hence, the application of TEA-CIFunciona can improve service provision for ASD, as it explicitly examines the components of functioning that are often left out of traditional health care provision. TEA-CIFunciona could help communicate functional information in a simple way (for example using an illustration shown in Figure 2), and therefore may encourage the pediatrician to collaborate in teamwork and adopt a social model to evaluate disability.

TEA-CIFunciona showed extremely varied school experiences, being very positive for some children and extremely negative for others. The latter was associated with negative attitudes of the teachers and problems in adopting effective individual educational strategies, such as anticipation pictures, calendars, or communication devices.

Again, TEA-CIFunciona helped us identify the main environmental barrier in our national context. We found that augmentative communication technology was not commonly used. The devices were unknown to many of the parents and therapists but highly valued by those who did use them. Although evidence is limited, different studies have reported on the possible benefits of augmentative communication devices for children with autism. Therefore, the lack of use or restricted use was considered as a barrier, mainly in children with important verbal language impairment [25].

### 4.2. User Instructions TEA-CIFunciona

**Figure 3:**
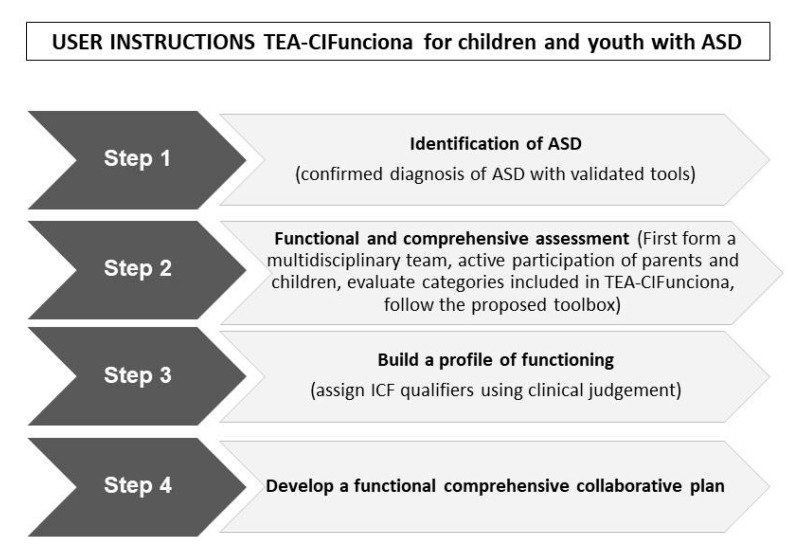
User instructions TEA-CIFunciona version 1.0.

To facilitate adoption and implementation of TEA-CIFunciona in clinical practice, we propose simple user instructions (Figure 3). Briefly, (1) to apply TEA-CIFunciona children and youth have to have a confirmed diagnosis of ASD, using validated diagnostic tools; (2) a multi-disciplinary team with active collaboration with parents and/or caregivers and children with ASD—when possible should participate in the assessment process, a proposed toolbox of items is suggested to address each ICF category contained in TEA-CIFunciona version 1.0; then (3) build a profile of functioning to summarize the findings of the assessment and highlights functional strengths and needs, along with environmental barriers and facilitators; finally (4) develop a functional comprehensive collaborative plan of intervention.

### 4.3. Contributions of TEA-CIFunciona to Clinical Settings

This study proposes a new culturally sensitive ICF-based tool for clinicians working in the field of childhood-onset disabilities, contributing to the few pediatric ICF tools available today [13]. The application of TEA-CIFunciona could facilitate comparison of outcomes and results of intervention across the country and hopefully in other countries in Latin-America. However, clinical experience as well as training in the use and the ICF language, are needed.

### 4.4. Additional Considerations and Contributions to the International Community

Asking about parents’ concerns and opinions also forces us to reconsider what the goals of treatment should be, who should set the priorities for a child, how to assess quality of care and support services, and even when to discontinue treatments. As described by Campos et al. in a recent qualitative study applying the ICF in a pediatric Latin American setting, it is crucial to incorporate parents’ perspective when describing functional needs and setting goals for interventions, as parents are the ones who live and experience the main functional limitations of their children [26].

To our knowledge, this study describes the first attempt to operationalize the content of the ICF Core Sets for ASD in a pediatric clinical setting, as such; the multi-step methodology applied for developing TEA-CIFunciona may encourage the international community to replicate our effort in different cultural settings around the globe, and identify the most relevant items or tools to assess the content of the ICF Core Sets for ASD in their countries.

In addition, by providing evidence of functional needs and environmental barriers TEA-CIFunciona has the potential to guide health policies and may be useful guiding data collection at a regional or global level, facilitating comparisons across settings, guiding resource allocation, professional training, and capacitation. Overall, TEA-CIFunciona proved to be a practical framework and guideline for comprehensive assessments of ASD, although the time necessary for the interview may be a limiting factor.

### 4.5. Limitations

The translation of the scores of different grading scales into the ICF qualifiers posed several challenges. It is important to note that there is no gold standard that we could follow, currently, there are no rules guiding how to translate clinical information into ICF qualifiers in pediatric populations. Other studies applying the ICF in pediatric populations have used clinical judgement [10,22]. Our process required adjustments and modifications until the final guidelines for assigning the qualifiers were proposed. The lack of standardized tools to evaluate some of the categories, mostly those related to environmental factors, may be an additional limitation.

Finally, the selection of categories from the comprehensive ASD Core Set may have left out specific aspects that would be important in the evaluation of some children with ASD. Further revisions and larger studies across the country are needed.

## 5. Conclusions

In conclusion, implementation of TEA-CIFunciona version 1.0 is feasible and standardizes the assessment of children with ASD in our country. The toolbox operationalizes the content of TEA-CIFunciona and facilitates building a profile of functioning of children with ASD. This study may encourage colleagues to adopt TEA-CIFunciona, systematize the evaluations of patients with ASD using a biopsychosocial approach, benefitting both the children and their families. Although these findings describe a specific population, they may shed light on issues that are common to other children with ASD, regardless of where they live [23].

### Future Steps

Regarding the next steps, it will be necessary to expand the use of this tool in other contexts, starting with community centers in Argentina and then in other Latin American countries with similar cultural background to identify common barriers and facilitators, to obtain a more representative functional data of this population. This could help to optimize delivery of health and rehabilitation services, to contribute to evidence-based policies, and to respond most adequately to children’s and family’s needs. Finally, we expect that after ongoing clinical application of TEA-CIFunciona version 1.0 in different settings, and based on feedback and lessons learned, future revisions will be required to include updates and recommendations not only from the professionals and caregivers’ perspectives on daily functioning but from the children and youth with ASD perspectives as well.

## Figures and Tables

**Table 1:** TEA-CIFunciona version 1.0 and proposed toolbox.

Content of TEA-CIFunciona (*n* = 32 ICF Categories)		Item/s Used to Assess Content	Alternative Item/s and Tools to Assess the Content of Each Category (Based on Availability of Tools)			
Body Functions (b)						
b117	Intellectual functions	CAT/CLAMS–Cognitive domain *	WPPSI *	S. Binet		
b125	Dispositions and intra-personal functions	VAS (parents) *				
b134	Sleep functions	VAS (parents) *				
b140	Attention functions	VAS (parents) *				
b156	Perceptual functions	VAS (parents) *				
b1670	Reception of language	VABS (Subdomain receptive) *	CELF	GARDNER	PLS	CLAMS (CD receptive)
b1671	Expression of language	VABS (Subdomain expressive) *	CELF	GARDNER	PLS	CLAMS (CD expressive)
b7602	Coordination of voluntary movements	VAS (parents) *				
b7652	Tics and mannerisms	ADI-R (item 77) *	ADOS (ítem: D2 module 2,3 ó 4)			
b7653	Stereotypies and motor perseveration	ADI-R (item 78) *				
Activities and Participation (d)						
d110	Watching	ADI-R (item 50) *				
d115	Listening	CARS (item 8) *				
d130	Copying	CARS (item 2) *				
d155	Acquiring skills	VABS * (Domain Daily Living Skills)				
d250	Managing one’s own behavior	CARS (item 6) *				
d330	Speaking	Observation/Interview *				
d335	Producing nonverbal messages	ADI-R (42, 43, 44 and 45) *	ADOS 2 (module 2 A7)			
d350	Conversation	ADI-R (ítem 35) *	ADOS 2 (module 2 A5)			
d530	Toileting	VAS * (parents)				
d550	Eating	VAS * (parents)				
d720	Complex interpersonal interactions	VABS * (Subdomain Interpersonal Relationships)				
d7500	Informal relationships with friends	PEDSQL* (Social Functioning)				
d815	Preschool education	VAS * (parents)				
d820	School education	VAS * (parents)				
d920	Recreation and leisure	VABS * (subdomain Leisure Time)				
Environmental Factors (e)						
e125	Products and technology for communication	Self-developed question *				
e310	Immediate family	Family Apgar *				
e355	Health professionals	VAS *				
e430	Individual attitudes of people in positions of authority	VAS *				
e5502	Legal policies	Self-developed question *				
e555	Associations and organizational services, systems and policies	VAS *				
e5800	Health services, systems and policies	Self-developed question *				

* Denotes item or items used to assess the content of each category included in TEA-CIFunciona, the items were selected from standardized measurements. As shown, some categories were assessed using VAS or self-developed questions. First column describes the toolbox used in this study, the second column proposes alternative options to assess each category. **CAT/CLAMS:** Clinical Adaptive Test/Clinical Linguistic and Auditory Milestone Scale, **ADI-R:** Autism Diagnostic Interview-Revised, **ADOS-2**: Autism Diagnostic Observation Schedule-2, **WPPSI**: Wechsler Preschool and Primary Scale of Intelligence, **CARS:** Childhood Autism Rating Scale, **VABS**: Vineland Adaptive Behavior Scales 2, **CUD**: Unique Disability Certificate, **VAS**: Visual Analog Scale, **PedsQL**: Pediatric Quality of Life Inventory, **S. Binet:** Stanford-Binet Intelligence Scales, **Gardner:** Test de figura/palabra receptivo y expresivo Gardner, **PLS:** Preschool Language Scale, **CELF-4:** Clinical Evaluation of Language Fundamentals 4, **Family APGAR:** Adaptability, Partnership, Growth, Affection, and Resolve.

**Table 2:** Characteristics of the sample using TEA-CIFunciona version 1.0.

Characteristics of the Children with ASD	
Sample size	100
Age in months. Median (range)	89 (36; 192)
Age < 6 years	39% (39)
Age 6–16 years	61% (61)
Age of ASD diagnosis. Median (range)	42.5 (20;112)
Gender % (n)	Boys 81% (81)
	Girls 19% (19)
UBN (Unsatisfied Basic Needs)	10% (10)
Severity level (DSM-5) I Requiring support II Requiring substantial support III Requiring very substantial support	30% (30) 42% (42) 28% (28)
Language	Yes 67% (67)
	No 33% (33)
Attending school	Yes 97% (97)
	No 3% (3)
Associated medical conditions *	69% (69)
Sleep disorder	32% (22)
Obesity	20% (14)
Genetic syndrome	10% (7)
Chronic disease	5.8% (4)
Epilepsy	2.8% (2)
Associated developmental disorder **	70% (70)
Intellectual Disability/GDD	Yes 42% (30)
	No 12.8% (9)
	Not evaluated 61% (of the total sample)
Developmental Coordination Disorder	17% (12)
Anxiety	14% (10)
Language disorder	12.8% (9)
Behavioral disorder	8.5% (6)
ADHD (Attention Deficit Hyperactivity Disorder)	8.5% (6)
Hearing Impairment	5.7% (4)
Learning disorders	4.3% (3)

* Associated medical condition: The values expressed in % were calculated from the 69% (the sample with an associated medical condition). The same child may have more than one associated medical condition. ** Associated developmental disorder: The values expressed in % were calculated from the 70% (the sample with an associated developmental disorder). The same child may have more than one associated developmental disorder.

**Table 3:** Frequency (%) of impairment, limitations/restrictions, barrier or facilitator in body functions, activities and participation and environmental factors, (*n* = 100 children with ASD).

**Category**	**Body Functions**	**Qualifier 0 (No Problem)**		**Qualifier 1 (Mild Problem)**		**Qualifier 2 (Moderate Problem)**	**Qualifier 3 (Severe Problem)**	**Qualifier 4 (Complete Problem)**		**Qualifier 8** **(No Specified)**		**Qualifier 9 (Not Applicable)**
b117	Intellectual functions	9		12		10	7	1		61		-
b125	Dispositions and intra-personal functions	5		34		52	8	1		-		-
b134	Sleep functions	66		12		13	9	-		-		-
b140	Attention functions	20		49		19	12	-		-		-
b156	Perceptual functions	33		35		17	10	5		-		-
b1670	Reception of language	16		39		32	11	2		-		-
b1671	Expression of language	6		21		56	14	3		-		-
b7602	Coordination of voluntary movements	58		22		16	3	1		-		-
b7652	Tics and mannerisms	37		29		27	7	-		-		-
b7653	Stereotypies and motor perseveration	19		38		35	8	-		-		-
**Category**	**Activities and Participation**	**Qualifier 0**		**Qualifier 1**		**Qualifier 2**	**Qualifier 3**	**Qualifier 4**		**Qualifier 8**		**Qualifier 9**
d110	Watching	13		51		31	5	-		-		-
d115	Listening	19		45		29	7	-		-		-
d130	Copying	33		43		20	4	-		-		-
d155	Acquiring skills	10		46		36	7	1		-		-
d250	Managing one’s own behavior	10		31		52	5	2		-		-
d330	Speaking	41		26		16	11	6		-		-
d335	Producing nonverbal messages	27		36		24	11	2		-		-
d350	Conversation	3		18		34	38	7		-		-
d530	Toileting	56		12		13	11	8		-		-
d550	Eating	31		22		28	11	8		-		-
d720	Complex interpersonal interactions	4		20		65	10	1		-		-
d7500	Informal relationships with friends	3		19		43	20	3		12		-
d815	Preschool education	24		8		4	2	4		-		58
d820	School education	36		6		4	5	7		-		42
d920	Recreation and leisure	1		29		53	14	3		-		-
**Category**	**Environmental Factors**	**Mild barrier (1)**	**Moderate barrier (2)**	**Severe barrier (3)**	**Total barrier (4)**	**No barrier/Facilitator (0)**	**Mild facilitator (+1)**	**Moderate facilitator (+2)**	**Severe facilitator (+3)**	**Total facilitator (+4)**	**No specified**	**Not appli-cable**
e125	Products and technology for communication	16	19	19	3	19	3	8	11	2	-	-
e310	Immediate family	-	1	-	-	3	13	32	44	7	-	-
e355	Health professionals (pediatrician)	-	5	8	1	18	15	24	22	-	7	-
e430	Individual attitudes of people in positions of authority (school authorities)	6	3	12	3	3	8	36	23	1	1	4
e550	Legal policies	-	-	3	-	-	-	-	97	-	-	-
e555	Associations and organizational services, systems and policies	2	2	-	-	49	8	11	13	1	14	-
e5800	Health services, systems and policies	2	1	13	8	-	15	19	42	-	-	-

## Data Availability

Due to privacy and confidentiality issues, we only share aggregated data in this study.

## Supplementary Materials

The following are available online at https://www.mdpi.com/article/10.3390/ijerph18073720/s1. Examples of translation strategies for ICF categories included in TEA-CIFfunciona version 1.0 for the components body functions, activities and participation and environmental factors.
