# Supplementary material for: Developing a Culturally Sensitive ICF-Based Tool to Describe Functioning of Children with Autism Spectrum Disorder: TEA-CIFunciona Version 1.0 Pilot Study
Source: Int J Environ Res Public Health. 2021 Apr 2;18(7):3720. doi: 10.3390/ijerph18073720 (PMC8038152; doi:10.3390/ijerph18073720)
Supplement: Supplementary file 1 [file ijerph-18-03720-s001.pdf]

Supplementary Material S1 Examples of Translation Strategies

Category Included in TEA-CIFunciona	Tool	Translation into ICF Qualifier	Considerations
		**Functions**	
**b134**<br>(sleep functions)	VAS	The score was directly translated into the ICF qualifier	A questionnaire related to the category was used as a guide. Then parents were asked to choose a value from the VAS.

**0**	**1**	**2**	**3**	**4**
None Problem	Slight problem	Moderate Problem	Serious Problem	Complete Problem
0–4%	5–24%	25–49%	50–95%	96–100%

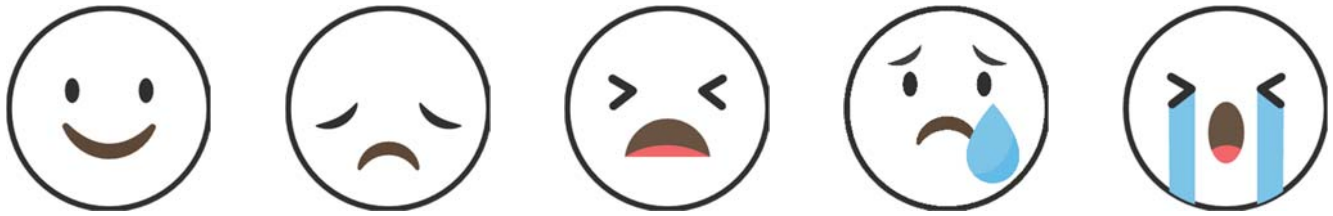

**b1671** (expression of language)	VABS (Subdomain receptive)	We translated the adaptive level using v-Scale into ICF Qualifiers	When the scoring was >12 the qualifier 0 was assigned; with a score 10–12, the qualifier assigned was 1; a score 6–9 received a qualifier 2; if a score 3–5 was given, the qualifier assigned was 3; and with score <3 a qualifier 4.
**b7653** (functions related to involuntary movements. Stereotypies and motor perseveration)	ADI-R (item 78)	Following Translational strategies, the professional rated item 78 and translated it into the ICF qualifier	When the scoring was 0, the qualifier 0 was assigned; with a score 1, the qualifier assigned was 1; with a score 2 a qualifier 2. If a score of 3 was given on the scale, the researcher decided by clinical judgment if the category represented a severe or complete problem.

(a)

	**Activities and Participation**		
**d155** (acquiring skills)	VABS (Domain Daily Living Skills)	We translated the adaptive level using Standard Score into ICF Qualifiers	The scoring was as follows: >86, the qualifier 0 was assigned; score 71–85, received a qualifier 1; with a score 51–70 a qualifier 2 was assigned; when the score was 35–50, the qualifier 3 was assigned; and a score <35 a qualifier 4.
**d250** (managing one's own behavior)	CARS (item 6)	The professional rated item 6 and translated it to the ICF qualifier following the Translational strategies	When the scoring was 1, the qualifier 0 was assigned; when the score was 2, the qualifier assigned was 1; score 3 received qualifier 2; and when score was 4, the researcher decided by clinical judgment if the category represented a severe or complete problem.
**d330** (speaking)	Observation/Interview	The professional rated the category following ADOS guideline	Non echoed phrases speech: 0<br>Echoed phrases, stereotyped utterances with other flexible spontaneous language: 1<br>Single words mostly: 2<br>No spoken language OR only speech without communicative intent: Up to 5 years, 11 months: 3– >6 years old: 4
**d815** (preschool education)	VAS	The score was directly translated into the ICF qualifier	A questionnaire related to the category was used as a guide. Then parents were asked to choose a value from the VAS.

0	1	2	3	4
No Difficulty	Mild Difficulty	Moderate Difficulty	Severe Difficulty	Complete Difficulty
0–4%	5–24%	25–49%	50–95%	96–100%

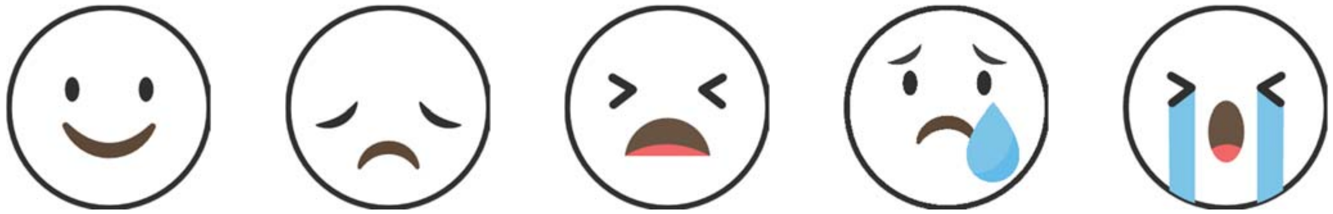

(b)

Environmental Factors			
**e125** (products and technology for communication)	Interview	The professional defined a facilitator or a barrier according to his or her clinical judgement	Levels of severity were expressed in % as defined in the ICF generic scale
**e355** (Individual attitudes of people in positions of authority)	VAS	The score was directly translated into the ICF qualifier	A questionnaire related to the category was used as a guide. Then parents were asked to choose a value from the VAS.

**−4**	**−3**	**−2**	**−1**	**0**	**+1**	**+2**	**+3**	**+4**
**Total Barrier**	**Severe Barrier**	**Moderate Barrier**	**Mild Barrier**	**No Facilitator/ no Barrier**	**Mild Facilitator**	**Moderate Facilitator**	**Substantial Facilitator**	**Complete Facilitator**

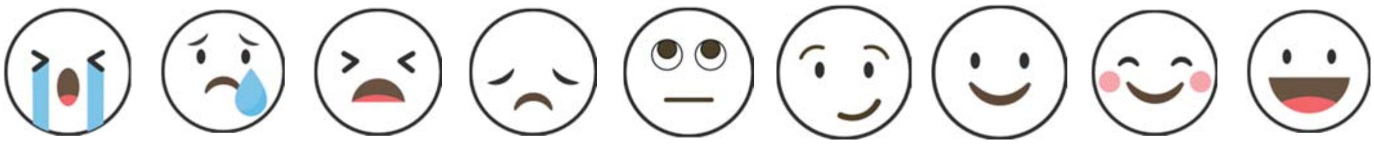

(c)
